# X-ray reflectivity from micrometre-scaled surfaces using nanobeams

**DOI:** 10.1107/S1600576725008179

**Published:** 2025-10-24

**Authors:** Vedran Vonk, Steffen Tober, Steven J. Leake, Breno Rabelo Coutinho Saraiva, Lisa Randolph, Arti Dangwal Pandey, Thomas F. Keller, Hans-Georg Steinrück, Andreas Stierle

**Affiliations:** a Centre for X-ray and Nano Science CXNS Deutsches Elektronen-Synchrotron DESY Notkestraße 85 22607 Hamburg Germany; b Institute for a Sustainable Hydrogen Economy (INW), Forschungszentrum Jülich GmbH, Marie-Curie-Straße 5, 52428 Jülich, Germany; c ESRF, The European Synchrotron, 71 Avenue des Martyrs, CS40220, 38043 Grenoble Cedex 9, France; d RWTH Aachen University, Institute of Physical Chemistry, Landoltweg 2, 52074 Aachen, Germany; e Physics Department, University of Hamburg, 20355 Hamburg, Germany; Institut de Recherche sur les Céramiques, France

**Keywords:** X-ray reflectivity, X-ray nanobeams

## Abstract

Sample and diffractometer alignment for grazing-incidence X-ray measurements become ever more crucial once the beam and surface area of interest reach the nanometre scale. Here we show how a point of interest on a surface can be kept in the beam while measuring X-ray reflectivity, even if it is not in the centre of rotation, either because of systematic errors or additional unwanted angle-dependent sample movement. This can be achieved by a 1D trajectory scan varying the angle of incidence (θ), the detector angle (2θ) and the position of the sample along one axis in the scattering plane. As an example, we show the results of X-ray reflectivity measured from a 10 × 10 µm Au island using a 90 nm beam. Data analysis is presented which considers the angle-dependent X-ray beam footprint illuminating both the Au island and the surrounding support.

## Abstract 2

X-ray reflectivity data collection using a nanobeam and model fitting of small micrometre-sized areas on a substrate are presented.

## 1. Introduction

X-ray reflectivity (XRR) can be used to deduce the interfacial electron-density profile near a sample’s surface, laterally averaged over the illuminated area and projected onto the area’s normal direction (James, 1958 ▸; Tolan, 1999 ▸; Als-Nielsen & McMorrow, 2011 ▸). Since the measured signal originates from an illuminated area, an averaging over local fluctuations is performed. This can have an effect on the deduced electron-density profile through the details of the surface roughness, which contains both height and lateral components (Sinha *et al.*, 1988 ▸), *i.e.* the details of the typical lateral lengths over which the surface undulates and how these affect the intensity of the specularly reflected X-rays (Press *et al.*, 1996 ▸). In general, near-atomic-scale details can be uncovered, which has led to XRR becoming a widespread method to determine thicknesses of thin films and multilayers and their quality, and also for technological applications.

Whereas conventionally XRR is used to characterize surface areas in the range of mm^2^–cm^2^, the advent of X-ray nanobeams can potentially reduce that to the order of µm^2^ and even smaller. Micrometre-scale characterization is necessary for a wide range of materials with a heterogeneous structure, for which the use of large X-ray beams would average out these local deviations. Two types of samples may be envisaged: those with an extended face and those with nano-scaled facets, such as nano-objects, either isolated or dispersed on a support. In the former case, where typical examples are surfaces of polycrystalline materials or stepped surfaces (de Poel *et al.*, 2014 ▸), the sample surface can be scanned and spatially resolved XRR data may be acquired. Here, the spatial resolution is largely limited by the projected beam’s footprint on the surface. In the latter case, where a typical example would be a faceted nanoparticle, the surface may be so small that it is illuminated completely while recording an XRR curve and thus its size affects the obtained spatial resolution. Similar experiments, but using high-angle Bragg–Brentano-type geometries, have already been used for Bragg coherent diffraction imaging (BCDI), thereby reconstructing the shape of single nanoparticles (Robinson *et al.*, 2001 ▸), mapping out domain structures in thin films (Schmidbauer *et al.*, 2017 ▸; Udovenko *et al.*, 2024 ▸) or recording facet scattering from single etch pits (Vonk *et al.*, 2018 ▸). By using grazing-incidence geometry, the possibility is envisaged of scanning a surface through the beam and obtaining local surface structure information, such as thicknesses of overlayers (XRR) or full crystallographic details through crystal truncation rods (Stubbs *et al.*, 2021 ▸).

A central isssue with scanning techniques is that one needs to be sure of where exactly the beam is illuminating the surface. This can be achieved by using structures well defined in size and pre-characterized by microscopy techniques, in combination with alignment procedures in the X-ray beam to enable the mapping on an absolute length scale. Here we describe alignment procedures using structures that are in the 10–100 µm size range, which matches the typical footprint of an approximately 100 nm beam illuminating the sample at the critical angle for total external reflection (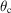 ∼ 0.1–0.2° at 10 keV). The lateral micrometre scale of the samples studied here is a first step towards potential future experiments targeting structures in the nanometre regime.

The following sections describe the effect of any misalignment between sample, beam and the diffractometer’s centre of rotation on the location of the footprint in the sample plane, given for grazing-incidence geometry. Two cases are described: a perfectly rigid instrument and when a particular type of (elastic) deformation takes place, which leads to an additional unwanted sample movement. Next, a description is given of an alignment procedure and the extracted trajectory to keep an area of interest in the beam, while measuring the XRR curve. Finally, the results of sample measurements and their analysis by a model fit are given.

## 2. Scattering geometry

**Figure 1:**
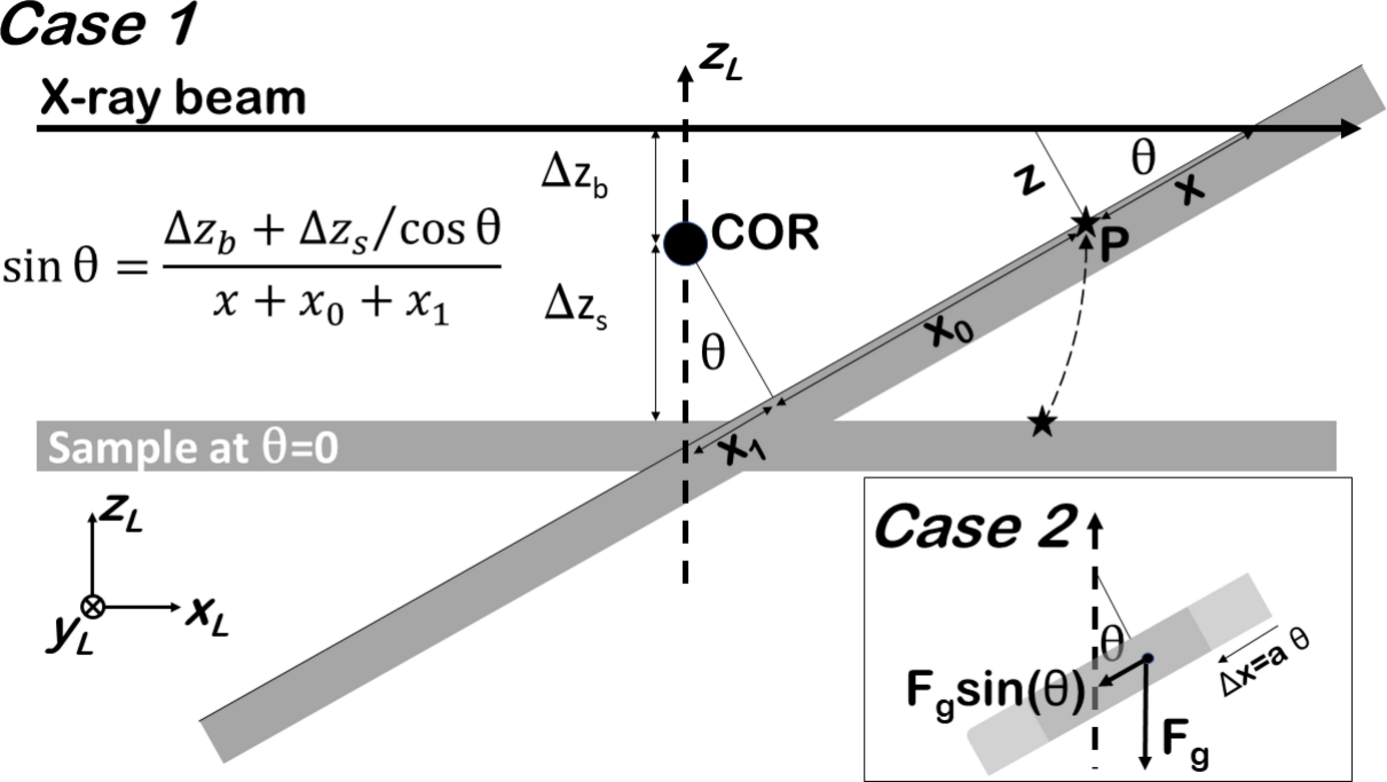
Schematic of the scattering geometry assuming a systematic error given by the sample and beam not coinciding with the COR. The X-ray beam is much smaller than the polished extended crystal face, from which X-ray reflectivity is to be determined. The angle of incidence (θ) is scanned and it is evaluated at which positions 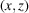 point P can be brought into the beam. The COR of the θ axis is indicated by the black marker and the (misalignment) offset distances of the beam and sample are given as 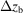 and 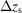, respectively. The origin of 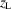 lies at the COR and its positive direction is indicated. Shown are the situations of a perfectly rigid set-up (Case1) and (in the inset) the assumption that the sample moves laterally with angle θ due to a change in the weight distribution (Case2), as indicated by the components of the gravity force (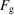) and as explained in the main text. The *y* axis is perpendicular to the drawing plane.

A reflectivity measurement is performed by rotating the sample in the beam and placing the detector to record the specularly scattered signal: a standard θ–2θ scan, with θ the angle between the surface and the beam, and 2θ the angle between the direct and reflected beams. The X-ray beam should pass through the rotation axes of the sample and detector (which are identical), and the point of interest (P) on the surface must also be placed in the centre of rotation and thus in the beam – a typical situation for a sample mounted on a diffractometer. For each instrument, specific protocols are usually available to align the sample in the beam and to define zero positions of the different circles (Hamilton, 1974 ▸). Alignment of the sample is done by performing a number of scans, placing it, as described above, as well as possible (Gibaud *et al.*, 1993 ▸). The precision with which these standard procedures need to be done depends on the beam and sample sizes and on the quality of the sample, *i.e.* how sharp the rocking curves are along the reflectivity ridge. When the X-ray beam has a size of micrometres or smaller, the alignment precision becomes more stringent and set-ups enabling such measurements are usually equipped with piezo motors (Leake *et al.*, 2019 ▸). The general scattering geometry in Fig. 1 ▸ shows schematically where the beam impinges on the surface with angle θ and the geometrically relevant distances around the centre of rotation (COR).

Here it is explicitly evaluated to what extent a non-ideal alignment of rotation axes, beam and sample, leading to a possible non-concentric rotation, affects the XRR measurement: in particular, how exactly a small surface area remains in the beam. Even though an alignment procedure must ensure that the X-ray beam goes through the COR of the diffractometer circles, errors will be present. At best, these errors are as large as the instrument’s sphere of confusion (SOC), which represents the overall systematic error. However, there are many (mechanical) factors that contribute to the instrument’s SOC and most often values are stated that include all the circles. For XRR measurements, which are typically done using two circles over a rather limited angular range, the manufacturer’s stated value for the SOC is therefore an upper limit. In addition, by considering only the contribution of the instrument’s circles to the SOC, a possible contribution from the sample mounting, which typically consists of a hexapod, piezo table and sample environment, is neglected. These issues are investigated in two ways. Case1 assumes a perfectly (mechanically) rigid instrument and sample mounting, and evaluates systematic errors in the alignment (SOC = 0). Case2 evaluates in addition the influence of a non-rigid instrument and/or sample mount (SOC ≠ 0), leading to a lateral dis­place­ment of the sample. This scenario is rationalized by considering the horizontal sample geometry, which means that the total weight distribution of the sample mount, which consists of a hexapod and a piezo scanner, changes with rotation around the θ axis. For small angular ranges it is not expected that this will lead to a large displacement along *z*; rather, the displacement will be mostly along *x*.

From the geometry as shown in Fig 1 ▸, it is evaluated how a point of interest (P), which lies in the surface plane, needs to be translated along either *x* or *z* to place it in the beam. The following expressions for *x* and *z* are obtained for Case1: 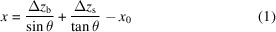
and, through 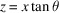, 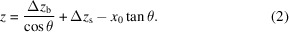
Here, 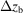 is the distance, along the laboratory *z* axis (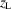), between the COR and the beam, and 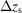 is the distance along 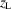 between the COR and the sample, when it is parallel to the beam. The angle between the beam and the surface is given by θ, and its zero point is defined as the surface and beam being parallel. In the case of perfect instrument and sample alignment, 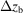 = 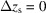. The point of interest lies at distance 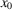 along the surface from the point (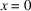) that intersects the *z* axis when the sample is parallel to the beam. An initial alignment procedure, which also ensures the 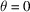 condition, should put the sample in the beam, after which the offset values 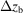 and 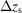 are (almost) equal but differ in sign (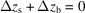). It is important to realize that, for a given angle θ, point P can always be brought into the beam by an infinite number of combinations of *x* and *z* values; in this sense, there is no unique way to align the sample to the required geometry. As soon as one fixes either the *z* or *x* positions of the sample, a unique trajectory for the other direction exists, as described by equations (1) and (2). Thus, by performing a trajectory scan using three coordinates θ, 2θ and one sample position (*x* or *z*) an XRR measurement can be performed. Such a scan is faster than a possible alternative: laterally scanning and mapping out the sample position at each θ–2θ position along the reflectivity curve in order to search for and find the area of interest in the beam.

Equations (1) and (2) are used when considering Case1 as described above. For Case2 the lateral position of the sample is also made a function of angle θ, which results in
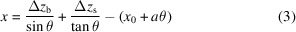
and 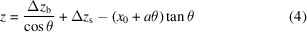
with *a* being a constant. Such a linear expression for the lateral movement is a simple first-order approximation, because the real behaviour is not known. In the geometry used here (discussed above), it is expected that the change (derivative with respect to angle θ) in the lateral component of the gravitational force exerted on the sample mount is the largest upon rotation of θ. Equations (3) and (4) then describe the situation when the sample’s *x* position is linearly proportional to 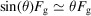 (when 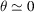), with 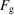 the gravitational force.

By evaluating the derivative and small-angle approximation of equation (1) (see Appendix *A*[App appa]), it is seen that *x* has a distinct non-linear behaviour with respect to angle θ and that its value can diverge when θ approaches 0. This can be understood by considering that, when the sample is not in the beam, for 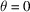 the surface is parallel to the beam and therefore would need to be translated infinitely along *x*. For the *z* direction, such a divergence does not exist, and the trajectory is, in first-order approximation, linear with θ [for 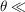 1°, see equation (8)]. In the more general case, both trajectories show an extremum, given by 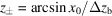 and 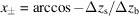. Depending on the exact values, these can also occur in the low-angle XRR regime, making it necessary to change the direction of the sample translation while performing the trajectory reflectivity scan.

There are different sources of errors involved, which will determine how accurately a particular point of interest can be aligned in the beam. An important aspect of the grazing-incidence geometry is that the beam projected onto the sample’s surface will lead to a footprint with length 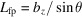, with 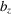 the height of the X-ray beam. This footprint gets larger with decreasing angle and ultimately will illuminate the sample over its entire length. It is then of interest to evaluate a possible error (ΔX) in the positioning of P in the centre of the illuminated area relative to the footprint’s length. In the case of the *x* direction, this is straightforwardly determined by the precision (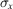) with which the sample can be aligned laterally. If we conservatively estimate this error to be 100 nm, which would represent the systematic error from reproducibly moving (piezo) motors, together with a beam having 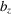 = 100 nm, then any misalignment will be within the beam’s footprint length: ΔX_*x*_ = 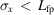. In the case of using *z* to position the sample, the evaluation needs to take into account the coupling with *x* using 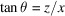. Then it follows that ΔX_*z*_ = 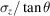. Normalizing it by the angle-dependent footprint leads to ΔX_*z*_ = 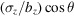, which shows that, in order to reduce the error to a fraction of the footprint size for small angles, the alignment precision must be much better than the beam size (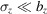).

Misalignment due to errors in angle of incidence can be evaluated using equation (6) (see Appendix *A*[App appa]). If we conservatively (upper limit) estimate any error in θ to be 0.05°, then it also follows that any of the sample’s lateral misalignment is still within the beam’s footprint. We note that a misalignment in the incident angle is typically negligible compared with the translational sample misalignment.

The diffractometer used for this study consists of rotation stages which allow for rotation around a certain axis and stepper motors. The calibration of their angular travel range is much more precise than that of the absolute position. As such, for the diffractometer used here we can neglect any non-ideal coupling between the θ and 2θ axes. For diffractometers that use several motions to set a certain angle, the situation can be quite different and such a non-ideal coupling can become crucial.

## 3. Experiment

By a lithography and lift-off process, micrometre-sized, isolated, square Au islands were fabricated on a Si wafer. The nominal thickness of the Au was chosen to be 10 nm. Results here are shown for island sizes of 100 × 100 µm and 10 × 10 µm. Another sample consists of an approximately 100 µm large Pt marker, which was written on a Mg–Al alloy substrate in the scanning electron microscope by local e-beam-assisted deposition from a Pt-containing precursor gas (Stierle *et al.*, 2016 ▸).

X-ray measurements were performed at beamline ID01 (Leake *et al.*, 2019 ▸) at the European Synchrotron Radiation Facility, France. The 9 keV X-ray beam was focused to 90 nm using Fresnel zone plates. A 2D hybrid pixel detector (pixel size 55 µm) was mounted approximately 1.5 m away from the sample. For the alignment scans, the integrated intensity in a region of interest (ROI) was taken. This ROI was centred around the pixel where the direct beam hits the detector with all diffractometer angles at zero; it was 12 pixels high in the scattering direction and 70 pixels wide, which results in an angular acceptance of 0.025° × 0.14°. Dry nitrogen, in combination with oxygen/air, was flushed over the sample’s surface throughout the measurements in order to reduce any radiation damage. The sample holder was mounted on an *xyz* piezo scanner, which sits on a hexapod. The mounting was such that the sample was lying horizontal in the laboratory frame, with the rotation axes for θ and 2θ along the *y* axis, as defined in this study. An alignment procedure, as described in the previous section, was carried out to keep a selected area in the beam. Coarse translations larger than about 0.1 mm are performed with the hexapod. Finer translations, below 0.1 mm, are done with the piezo scanner.

## 4. Results

**Figure 2:**
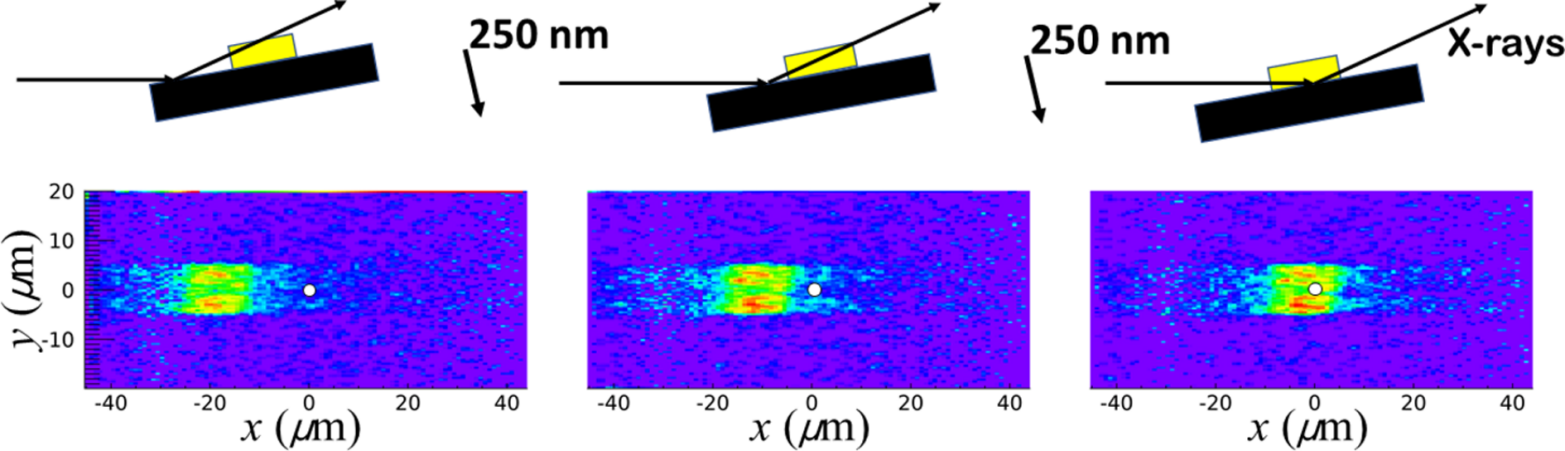
(Top) Schematic of the geometry and positioning of the sample, consisting of a Si wafer (black) and the Au island (yellow), in the beam. Shown are three different *z* positions of the sample, 250 nm apart, as indicated by the arrows. (Bottom) Corresponding intensity maps of the reflected signal at θ = 1.8° mapped in the surface *xy* plane. The white dots indicate the nominal position of the X-ray beam with the size corresponding to the nominal footprint along *x* of approximately 3 µm (that along *y* is exaggerated for clarity).

While the beam reflects off the surface at angle θ, the positioning of the sample is adjusted such that the Au island is in the beam. Fig. 2 ▸ shows several scans of the sample in the *xy* plane, while recording the reflected signal. The reflectivity of the Au island is substantially higher than that of Si, so a clear contrast is visible once the Au is in the beam. The scattering plane lies in the *xz* plane, and the incoming beam is along the *x* direction. Depending on its position along the *z* axis, the sample needs to be translated along *x* to put the Au island in the beam. For each 250 nm movement of the sample along *z*, the sample is translated approximately 8 µm along *x*, which fits the relation 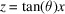, with θ = 1.8°. There is no adjustment needed along the *y* direction. These measurements show that, by translating along either *x* or *z*, the island can be put in the beam.

**Figure 3:**
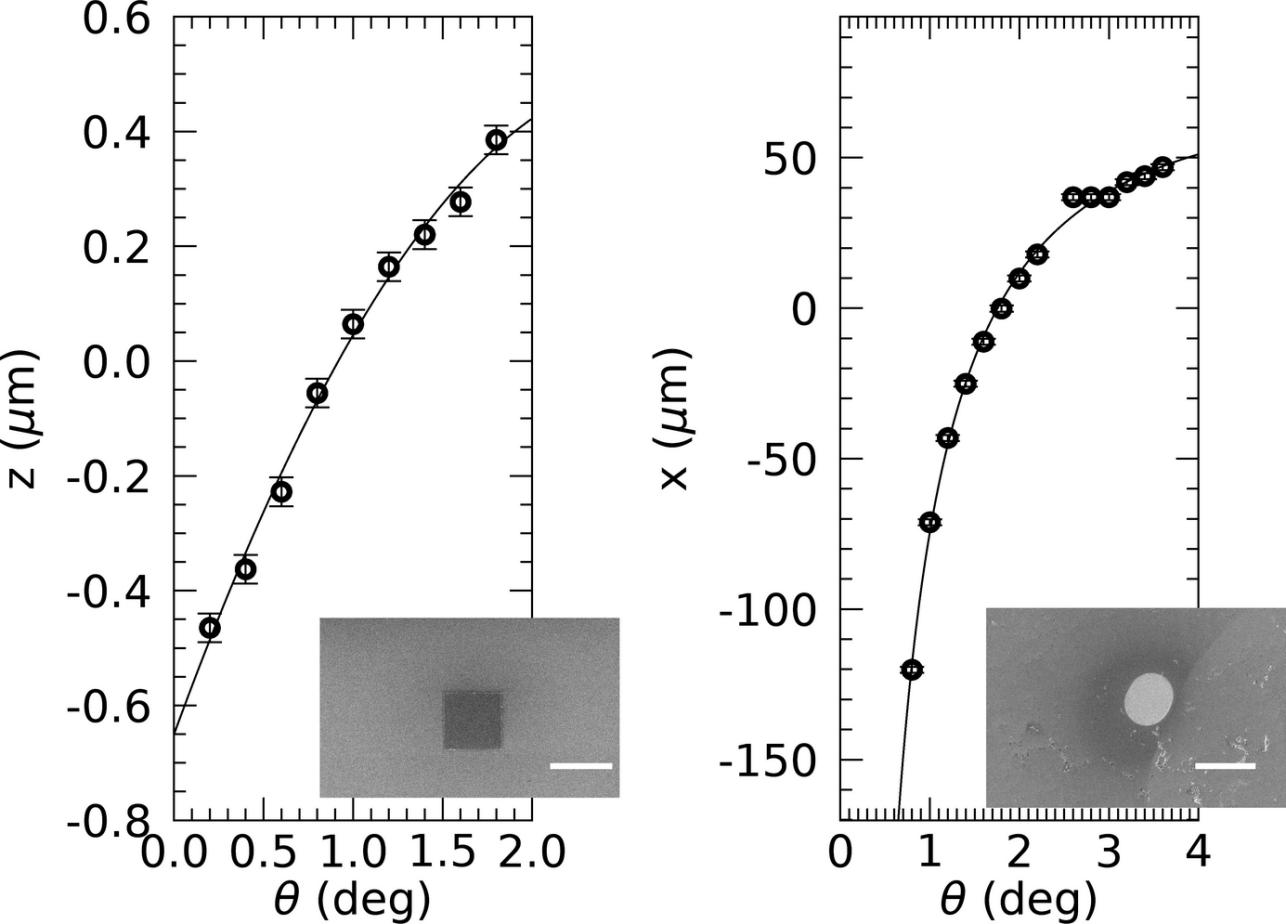
Results of sample alignment positions in grazing-incidence geometry. (Left) At different θ–2θ positions, the height *z* of the sample, which consists of a 10 × 10 µm Au island on a Si wafer, was scanned and the maximum reflected intensity was determined. The result of fitting equation (2) is shown as a solid line (black); fit parameters are listed in Table 1 ▸. The fit result using equation (4) is nearly identical and therefore not shown. The inset shows a scanning electron microscopy (SEM) image of the square Au island (scale bar represents 10 µm). (Right) Lateral sample positions (symbols) whereby an approximately 100 µm polycrystalline Pt marker was aligned in the beam at grazing angle θ and the Pt 111 (powder) Bragg reflection recorded. A fit of equation (1) is shown as a solid line (black); fit parameters are listed in Table 1 ▸. The fit result using equation (3) is nearly identical and therefore not shown. The inset shows an SEM image of the elliptical Pt marker (scale bar represents 100 µm).

Using similar alignment scans, Fig. 3 ▸ shows the results of tracking the sample’s *z* position at fixed *x* but varying θ, while keeping the 10 × 10 µm Au island in the beam. A fit of equations (2) and (4) to the found *z* positions is performed as well and the obtained parameters are listed in Table 1 ▸. The fits of equations (2) and (4) are found to be nearly identical.

In order to test the sensitivity and reproducibility with respect to slightly different initial sample orientations, like tilts, the same Au island was used in two more alignment runs. The results of these, together with those of another 100 × 100 µm Au island, are shown in Appendix *B*[App appb]. Fitting equation (2) while fixing 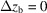 results in a linear fit curve with 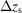 = −0.54 ± 0.01 µm. Such a linear relation describes less well the shape of the curve, but would still be a relatively good approximation up to θ ≃ 2°, where the footprint is about 3 µm and its centre position is around the border of the Au island. For even higher angles, the island would no longer be illuminated if the linear relation was used to extrapolate the trajectory. Also shown in Fig. 3 ▸ are the results of placing the Pt marker, deposited on a MgAl alloy, in the beam by lateral translation along *x*. These alignment scans were done at different incidence angles θ and keeping the detector fixed at twice the Bragg angle for the Pt 111 reflection, which forms a spotty powder ring. A model fit using equations (1) and (3) to the found positions is shown as well and the resulting parameters are listed in Table 1 ▸. The fits of equations (1) and (3) are found to be nearly identical.

**Figure 4:**
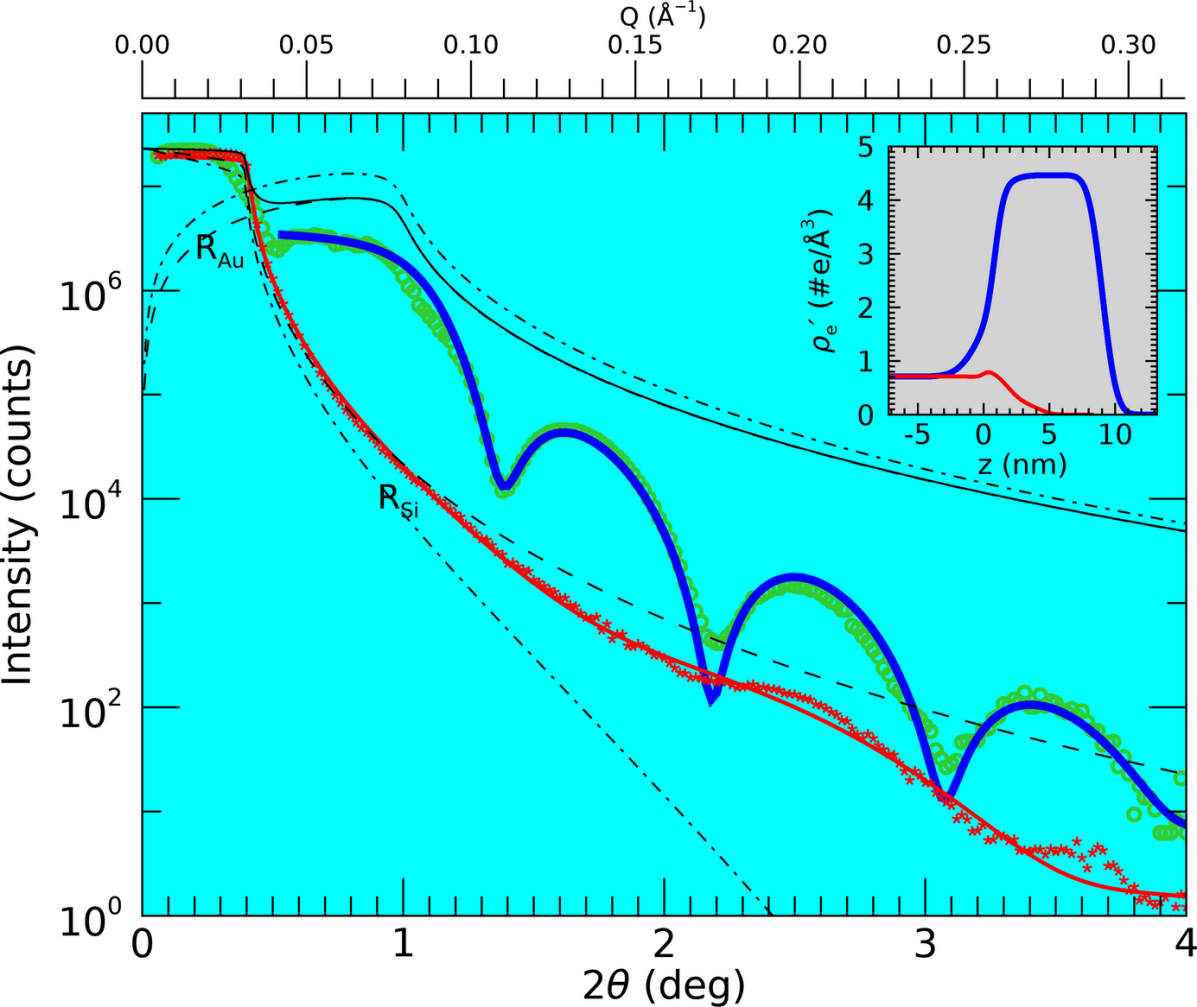
XRR from a 10 × 10 µm Au island on a macroscopic Si wafer and from the bare substrate. The measured data from the Au island (open green symbols) are shown together with simulated Fresnel curves for the interfaces Si/vacuum and Au/vacuum (dashed lines), thereby taking into acount that the 90 × 90 nm beam is only partly reflected from the island for small angles. Both Gaussian (dash) and Lorentzian (dash–dot) beam profiles are evaluated (see text for more details). Their combined reflectivity is shown as a black solid line. A fit to the data, starting from 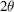
 = 0.55° omitting the lower angles, and using a slab model consisting of a Au layer on a Si substrate, is also shown (blue). The XRR curve for the bare substrate, measured just beside the Au island, is shown as well (red asterisks), along with a fit (red). The inset shows the resulting (electron) scattering density profiles as obtained from the fitting procedures for the Au island (blue) and the bare Si substrate (red).

Using the obtained *z* positions for the Au island sample, a trajectory scan along 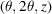 was performed and the XRR curve as shown in Fig. 4 ▸ obtained. For very small angles, the X-ray beam overspills the Au island and only a small part of it is intercepted and reflected. This represents the most common case in XRR measurements and a standard correction is applied (Gibaud *et al.*, 1993 ▸). At these small angles, the part of the beam that is not reflected by the island hits the bare substrate and is reflected by it. The 90 nm beam will start to overspill the 10 mm Si substrate for angles below approximately 0.5 mdeg, which is not captured by the present measurement. The recorded intensity is thus the sum of two reflected beams, which can be quantified as
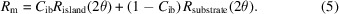
*R* is the reflectance, which is related to the measured intensity as 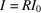, with 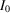 the intensity of the primary beam. The part of the beam, 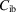, that is intercepted by the island with length 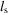 is given in Appendix *C*[App appc] for Gaussian and Lorentzian beam profiles, the effect of which is shown by simulation in Fig. 4 ▸.

In particular, the difference between the beam profiles becomes evident, with the Lorentzian having tails that extend much further out and which lead to a much more significant residual contribution from the substrate reflectivity compared with the Gaussian case over the whole angular range. In the case of a Gaussian beam profile the substrate contribution is negligible after a few times the critical angle of Si. Since the largest contribution to the data comes from the Au, the data starting just above the critical angle of Si (0.275°) are used and treated as if the sample consisted of a Au film completely covering the substrate. The resulting fit using the program *GenX* (Glavic & Björck, 2022 ▸) is shown in Fig. 4 ▸ and results in an 8.1 ± 0.5 nm island thickness. The density profile represents the scattering density 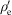 which is the electron density corrected for the dispersion at the given X-ray energy (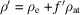, with 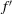 the dispersion and 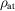 the atomic number density). For bulk Au 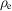 = 4.6 e Å^−3^ and at 9 keV 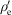 = 4.4 e Å^−3^ is obtained. The value obtained from the fit is 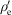 = 4.5 ± 0.2 e Å^−3^.

XRR was also measured just beside the Au island by translating the sample along the *y* axis. As seen in Fig. 4 ▸, the reflected intensity from the bare substrate is significantly lower than that from the Au island, which justifies omitting its contribution from the fitting procedure for the Au island data described above.

## 5. Discussion

The angle-dependent translational positions *x* or *z* found to align an object in the beam show non-linear behaviour as described very well by equations (1) and (2) (see Fig. 3 ▸). It is also evident that the distances over which the sample needs to be corrected along *x* are about 100 times larger than those in the *z* direction, which is a direct consequence of the 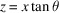 relation. This is important with respect to the stroke of the linear alignment axis used.

The larger the θ range over which the alignment trajectory can be performed, the better it can be determined. However, this range depends on the type of signal, its scattering contrast (signal-to-noise ratio) and the geometry. The intensity of the specularly reflected X-rays decays exponentially with increasing angle, resulting in measurable signals up to θ = 2° for the Au island studied here. This range is approximately twice as large for the powder-like Bragg signal from the Pt marker (see Fig. 3 ▸). Although this might indicate that the latter type of alignment would be better, there is no need to extend the XRR beyond the angle from where a measurable signal can be obtained. The optimal strategy will depend on the type of data required and on the sample details like scattering power and its quality.

The resulting fit parameters in combination with their estimated standard deviation (e.s.d.) suggest systematic deviations and correlations. Several alignment runs of the same 10 × 10 µm Au island give different results for 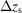 and 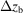. For Case1, the obtained values are roughly within 2 e.s.d. from each other, but the rather large range of approximately 1 mm suggests that the fit is not very sensitive to these values. In addition, diffractometer alignment is performed in a rigorous way, including the use of an optical microscope, and is expected to have an accuracy of the order of several µm. The values for 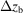 and 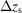 obtained in Case2 do indeed indicate that the instrument is aligned accurately, but their errors also show that the fit is not particularly sensitive to these values. A much more robust result is the sum 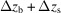, which for all cases results in a value of a few µm and represents the mis­alignment of the sample in the beam (and includes the residual instrument alignment error). These results suggest that the presented method cannot be used for adjustments to the alignment of the diffractometer, but that it does allow a small object to be kept in the beam. Furthermore, it seems that the large values obtained for 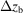 and 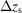 in Case1 are the result of the distinct non-linear shape of the curve: with small values, close to those expected, the fits are linear and give a worse fit. This points to Case2, although having a (nearly) identical fit, being a better description of the real behaviour of the instrument and sample mounting.

In the case of the Au island (Fig. 4 ▸), it is shown that, by neglecting the reflected signal from the simultaneously illuminated bare Si substrate, a meaningful fit can be obtained. The approximately 8 nm island thickness obtained corresponds well to the targeted value of approximately 10 nm. The density obtained from the fit corresponds within the error very well to the value for dense bulk Au. The fitted density profile obtained from the bare Si substrate (see the inset of Fig. 4 ▸) includes an approximately 3 nm thin surface region with a density close to that of pure Si and SiO_2_ (Awaji *et al.*, 1996 ▸). Still, some features of oscillations present in the data are not well reproduced, most likely due to organic residue from the sample preparation (Steinrück *et al.*, 2014 ▸), but it is beyond the scope of the current study to further analyse the lithography-prepared Si surface.

The beam size together with the details of its profile lead to a characteristic step at the critical angle of the substrate (see Fig. 3 ▸). The more extensive the beam’s tails, the larger the step. Simulation using a Lorentzian beam profile does not reproduce the measured intensity drop, which suggests that the real beam profile exhibits even more pronounced tails. Another explanation for the large drop might be that not all X-rays hitting the Au island are reflected (*R* < 1). This might be due to an angle-dependent effect where the effective beam divergence changes when only part of it is intercepted by the micrometre-scaled surface, possibly in combination with small-angle and/or off-specular scattering, which reduce the intensity of the specularly reflected beam. Here, the details of a possible coherent (small-angle) diffraction pattern from the island, consisting of rotated internal grains, may also play a role (Keller *et al.*, 2023 ▸).

## 6. Conclusions

The parametrization introduced in Section 2 is well suited for defining a trajectory for performing θ–2θ-type scans and translating the sample along one axis in the scattering plane, thereby keeping it within the beam’s footprint length at the same position on the surface. The grazing-incidence geometry results in the translation mostly along the beam (here called *x*) being about 100 times larger than that mostly perpendicular (*z*) to it. This is important in relation to the stroke and resolution of the used positioning axis. From the scatter in the numerical values obtained from fits when assuming a perfectly rigid instrument (Case1) or an additional lateral sample movement (Case2) or when slightly different sample tilts are used, it can be concluded that the presented method is not accurate enough for correcting systematic instrument alignment errors. This behaviour can be explained by the existence of large correlations between the different parameters.

In the most general case, for small angles the X-ray-illuminated area will become larger than the ROI. This means that one needs to take into account the scattering from the area around it as well. For samples like the one used in this study, namely a well defined smooth island supported by a material with a much lower scattering contribution, it is shown that reflectivity from the support can be largely ignored. The results presented here show that one can reliably obtain local information about the thickness and density of overlayers with a lateral resolution of about 10 µm. The described challenges and solutions concerning the exact location of the beam on the sample will be helpful for experiments where the surface is scanned through the beam under grazing-incidence geometry, for example in the case of polished polycrystalline materials, thereby enabling surface grain mapping. 

## Figures and Tables

**Figure 5:**
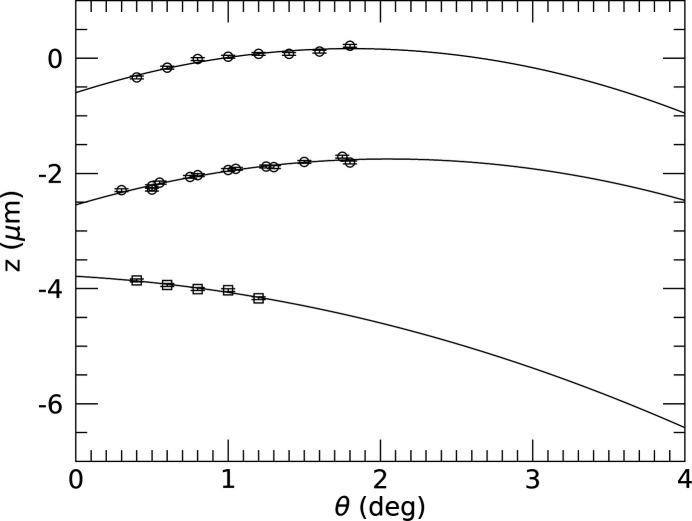
Alignment scans of Au islands. Shown are the results for the same 10 × 10 µm Au island (open circles) as shown in Fig. 3 ▸, but with different prior alignment settings, such as sample tilts. Also shown are the results of a 100 × 100 µm Au island (open squares). The results of fitting equation (2) are shown as solid lines; fit parameters are listed in Table 2 ▸.

**Table 1:** Results of fits to the measured sample positions as shown in Fig. 3 ▸ The type of structure and its size are indicated in µm. Parameters are those defined in equations (1)–(4), where the top values are for Case1 and the lower ones for Case2, which includes one extra parameter. Values and error bars are not rounded for a better discussion, as given in the main text. The fits for Case2 gave very large error bars for those parameters that are highly correlated and these are indicated as not available (n.a.). Experimental errors, which are directly related to the errors in the fit parameters, were taken as 25 nm for *z* and 1 µm for *x*.

	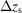 (µm)	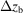 (µm)	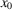 (µm)	*a* (µm per °)
Au 10 × 10				
Case1	−1048 ± 94	1047 ± 94	−49 ± 4	–
Case2	−0.37 (n.a.)	−0.29 (n.a.)	−49 ± 2	9 (n.a.)

Pt 65 × 80				
Case1	−277 ± 87	274 ± 87	−105 ± 3	–
Case2	−2 (n.a.)	−1 (n.a.)	−105 ± 5	2 (n.a.)

**Table 2:** Results of fitting sample positions The type of structure and its size are indicated in µm. Parameters are those defined in equations (1)–(4), where the top values are for Case1 and the lower ones for Case2, which includes one extra parameter. Values and error bars are not rounded for a better discussion, as given in the main text. Experimental errors, which are directly related to the errors in the fit parameters, were taken as 25 nm for *z* and 1 µm for *x*.

	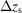 (µm)	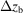 (µm)	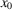 (µm)	*a* (µm per °)
Au 10 × 10				
Case1	−1533 ± 316	1532 ± 316	−48 ± 6	–
	−1241 ± 91	1241 ± 91	−53 ± 4	–
Case2	−0.32 (n.a.)	−0.27 (n.a.)	−48 ± 3	13 (n.a.)
	−0.31 (n.a.)	−0.24 (n.a.)	−44 ± 2	11 (n.a.)

Au 100 × 100				
Case1	−824 ± 440	824 ± 440	9 ± 15	–
Case2	0.24 (n.a.)	0.00 (n.a.)	9 ± 7	7 (n.a.)

## Data Availability

Research data are available upon request from https://doi.esrf.fr/10.15151/ESRF-ES-1824853946.

## Appendix A Approximations

The partial derivatives of equations (1) and (2) with respect to the angle of incidence θ are

These derivatives are used for error propagation analysis. In the case of exploring the linearity in the small-angle regime , equations (1) and (2) become

## Appendix B Sample positioning

Here the results of further positioning alignment are given. Fig. 5 ▸ shows the measurements and the fit results are listed in Table 2 ▸. The θ range is extended beyond the measurement points to better show the presence of a maximum in the trajectory, as discussed in Section 2.

## Appendix C Intercepted beam

Given a certain beam profile , the reflecting structure may intercept only part of it. One then needs to calculate

where  is the total intensity in the beam and  represents the boundaries between which the X-ray beam illuminates the sample. Calculating for a Gaussian, this becomes

with  the height of the beam expressed as the full width at half-maximum (FWHM). In the case of a Lorentzian profile, equation (9) results in

again with the beam height  expressed as the FWHM value.
